# Experimental characterization of bending effects for solid and hollow dielectric waveguides at V-band

**DOI:** 10.1038/s41598-021-00187-9

**Published:** 2021-10-19

**Authors:** Thanh-Luan Vu, Stéphane Barlerin, Yves Stricot, Ronan Sauleau, Mauro Ettorre, David González-Ovejero

**Affiliations:** 1 UMR 6164, IETR (Institut d’Electronique et des Technologies du numéRique), CNRS, Université de Rennes, 35000 Rennes, France; 2 APTIV, 28230 Épernon, France

**Keywords:** Electrical and electronic engineering, Mechanical engineering

## Abstract

Mm-wave dielectric waveguides are a promising and low-cost technology for the transmission of ultra-high data rates. Besides the attenuation (losses) and group delay, the bending loss of the dielectric waveguides is also one of the key parameters to establish the capacity and energy efficiency of such wired links, when deployed in realistic scenarios. In this context, we report the experimental characterizations of bending effects for various solid and hollow commercially available dielectric waveguides at V-band (50–75 GHz). A wide-band transition has been designed to carry out the measurements using a Vector Network Analyzer (VNA) and extension modules. The measured results are in very good agreement with full-wave simulations. Our experimental results show an average bending loss of 1.46 dB over the entire V-band for the fundamental ${HE}_{11}^{y}$ mode of a PTFE solid dielectric waveguide (core diameter of 3.06 mm) with a 90° bending angle and 25 mm radius of curvature. This value rises up to 2.88 dB (or 3.25 dB) when bending radius is changed to 15 mm (or bending angle grows up to 140°). The measurements also show that the measured bending losses increase significantly for hollow dielectric waveguides, in particular when the inner to outer diameter ratio gets larger.

## Introduction

Dielectric waveguides have recently sprung up as a very promising alternative to copper cables and optical fibers in multi-gigabit wired data links at millimeter waves for automotive or data center applications^1^. Various types of dielectric waveguides made of plastic (mainly PTFE or PS) have been used in recent demonstrations. Among them, it is worth mentioning shielded (with metal^2^ or foam^3^) and non-shielded waveguides^4^ with different cross-sections (hollow circular, solid rectangular). Although metallic shielded dielectric waveguides have been shown to provide impressive data rates^2,5,6^, the link distance using such waveguides is limited by their high attenuation^2^ and large dispersion near their cut-off frequencies^5^. This is the reason why non-shielded or foam-shielded dielectric waveguides have been also investigated in recent years^3,4,7–10^. The well-known properties of dielectric waveguides^11^ have been recently exploited to maximize the link’s data rate or to reduce its power consumption^12–14^. In single-mode dielectric waveguides, the main factors that determine the maximum achievable data rate are the attenuation and dispersion of the propagating mode^12^, whilst multimode dielectric waveguides with minimum dispersion have been proposed at W-band and D-band^13^. However, the losses in these waveguides are significantly higher than in single-mode ones. In addition, the analysis and experimental validation of hollow dielectric waveguides have been presented^14^. However, these studies^12–14^ do not include the effect of bending the waveguides, which is a crucial aspect for an accurate link budget calculation in realistic scenarios. Thus, it is difficult to find and select the most suitable dielectric waveguides for mm-wave communication links. In this article, we present the experimental characterization of bending effects at V-band for a solid circular dielectric waveguide and for hollow circular waveguides with different inner and outer diameters. The chosen dielectric waveguides can be easily purchased and can exploit two orthogonally-polarized modes for full-duplex operation or to double the data rate. In order to measure these waveguides, a specific transition from metallic circular waveguides to dielectric waveguides has been realized at V-band. The selected waveguides were measured for the two polarizations of the fundamental mode (${HE}_{11}^{x}$ and ${HE}_{11}^{y}$), for different bending angles (45*°*, 90*°*, 120*°* and 140*°*) and for two representative bending radii (25 mm and 15 mm). The bending losses are extracted from measured S-parameters and compared with simulation results. Based on the obtained values, we discuss the influence of bending on solid and hollow dielectric waveguides as well as its impact on link budgets using such transmission lines.

## Results

### Circular dielectric waveguides

Table 1 provides the geometrical details of the studied dielectric waveguides. All samples are made of PTFE with *ε*_*r*_ = 2.03 and tan*δ* = 0.0003 at 60 GHz. The waveguides present a mono-modal propagation of the fundamental ${HE}_{11}$ mode at V-band, since their cutoff frequencies for the first higher order mode (${TE}_{01}$- Transverse Electric) are higher than 75 GHz. Due to size tolerances, it is hard to get solid and hollow dielectric waveguides having exactly the same cutoff frequencies. However, among the samples shown in Table 1, the cutoff frequencies for the first higher order mode of waveguides SL and R3 or those of waveguides R1, R2 and HL1 are very close. This allows to compare bending effects between waveguides SL and R3 (or between R1, R2 and HL1).

**Table 1:** Characteristics of the measured circular dielectric waveguides (OD: outer diameter, ID: inner diameter).

	Type	Cross-section	Material	Measured size (mm)	ID/OD	Cutoff frequency (GHz)		Provider
						${HE}_{11}$	${TE}_{01}$	
SL	Solid		PTFE^15^, *ε*_*r*_ = 2.03, tan*δ* = 0.0003	OD = 3.06	–	0	75	Polyfluor
HL1	Hollow			OD = 2.94, ID = 1.10	0.37		80	
HL2	Hollow			OD = 2.96, ID = 1.50	0.51		84	
R1	Hollow			OD = 2.92, ID = 0.50	0.17		79	Reichelt Chemietechnik GmbH
R2	Hollow			OD = 2.96, ID = 1.10	0.37		79.56	
R3	Hollow			OD = 4.00, ID = 3.00	0.75		75.77	

**Figure 1:**
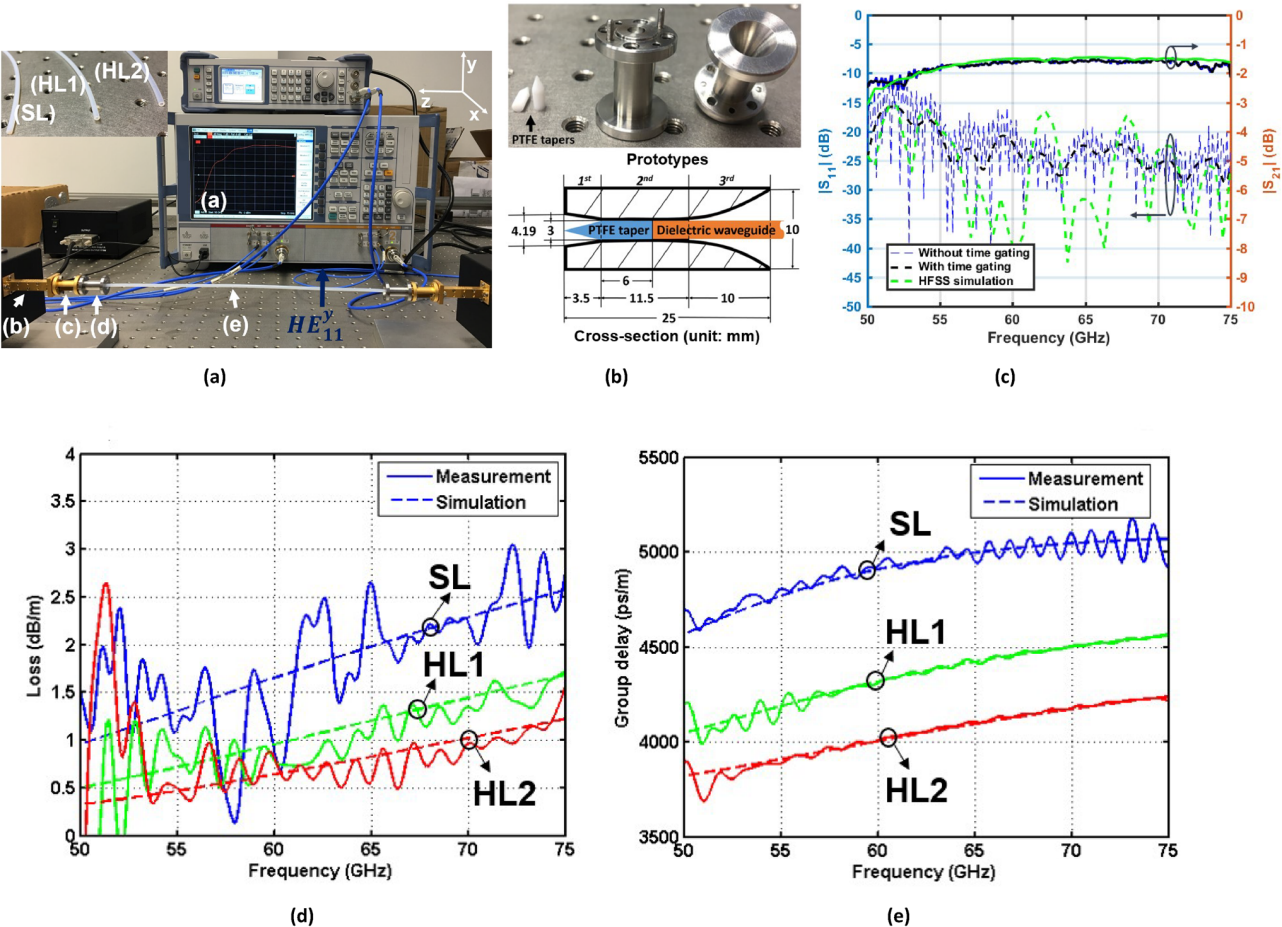
Experimental characteristics of measured straight dielectric waveguides for the ${HE}_{11}^{y}$ mode. **(a)** Experimental setup including VNA (1), extension modules with WR-15 output (2), WR-15-to-circular waveguide transition (3), circular waveguide-to-dielectric waveguide transition (4) and circular dielectric waveguide under test (5). **(b)** Transition from circular waveguide to dielectric waveguide. **(c)** Measured and simulated S-parameters for solid waveguide SL with a length of 306 mm in back-to-back configuration. **(d,e)** Measured and simulated attenuation and group delay of the solid (SL) and hollow waveguides (HL1 and HL2), respectively. An equivalent setup of this configuration was also simulated by Ansys HFSS^16^. Solid and dashed lines are measured and simulated results, respectively.

The selected dielectric waveguides (SL, HL1 and HL2) were measured under straight configuration for the ${HE}_{11}^{y}$ mode using the experimental setup shown in Fig. 1a. The dielectric waveguides are connected to a VNA with rectangular waveguide (RW) output, using a RW-to-circular waveguide (CW) transition and a broadband CW-to-dielectric waveguide transition. The metallic part of the latter module (shown in Fig. 1b) is formed by three sections, depicted in the section view in Fig. 1b. The first part is 3.5 mm long, and tapers the 4.19 mm diameter of the input circular waveguide to 3 mm. The second part has a constant 3 mm diameter and eases the insertion ofs the dielectric waveguides for measurements. The third part transforms the ${TE}_{11}$ mode of the dielectric loaded waveguide into the fundamental ${HE}_{11}$ mode of the non-shielded dielectric waveguide. Finally, on the side connected to the 4.19 mm circular waveguide, the transition includes a PTFE tapered insulation. This last element provides a good impedance matching between the hollow CW and the dielectric-filled one. The fabricated transition provides an input reflection coefficient lower than -20 dB and an insertion loss of less than 0.5 dB from 55 to 75 GHz. Figure 1c depicts the measured and simulated reflection and transmission coefficients of the back-to-back configuration using a 306 mm long solid waveguide SL. The measured magnitude of the transmission coefficient is in good agreement with full-wave simulations, carried out with Ansys HFSS. Nevertheless, at frequencies below 55 GHz, the measured *S*_21_ does not follow the trend of simulated results because of the sensitivity of the dielectric waveguides to assembly and handling during measurements. Such sensitivity is also considered the cause of the difference between simulated and measured |*S*_11_|. During measurements, the various parts of the back-to-back configuration are not perfectly aligned as in simulations. Unexpected reflections between these parts in measurements are thus expected. However, the obtained reflection coefficients (both in simulations and measurements) are considered acceptable with values lower than – 20 dB in the frequency range from 55 to 75 GHz. The attenuation and group delay of the dielectric waveguides labelled SL, HL1 and HL2 are calculated as follows^17^. The attenuation $\alpha$ in Np/m is computed from the measured magnitude of the S-parameters for two samples with different lengths (${L}_{1}$< ${L}_{2}$) as

1 $${e}^{-2\alpha({L}_{2}-{L}_{1})}=\frac{{|{S}_{11}|}_{{L}_{2}}^{2}+ {|{S}_{21}|}_{{L}_{2}}^{2}}{{|{S}_{11}|}_{{L}_{1}}^{2}{+|{S}_{21}|}_{{L}_{1}}^{2}}$$

In turn, the group delay for a waveguide of length $L$ is defined as the derivative of the ${S}_{21}$ phase with respect to the angular frequency as

2 $${GD}_{L}=\frac{\partial (\measuredangle {S}_{{21}_{L}})}{\partial \omega }$$

Note that the group delay in both transitions and the plastic waveguide is included in this calculation. Thus, we extract the group delay per unit length from the ${GD}_{L}$ computed for two samples with different lengths (${L}_{1}$< ${L}_{2}$) as

3 $${GD}_{DWG}=\frac{{GD}_{{L}_{2}}-{GD}_{{L}_{1}}}{{{L}_{2}- L}_{1}}$$

Figure 1d,e represent the attenuation and group delay per unit length calculated using Eqs. (1–3), respectively. The measured values fluctuate around the simulated ones. The solid SL, hollow HL1 and HL2 waveguides present across the whole V-band an average attenuation (group delay variation) per unit length equal to 1.82, 0.97 and 0.83 dB/m (500, 510.5 and 418 ps/m), respectively. Thus, these waveguides are significantly less lossy than metal-shielded dielectric waveguide E-tube^2^ (5 dB/m) in the band of interest.

### Bending configurations

**Figure 2:**
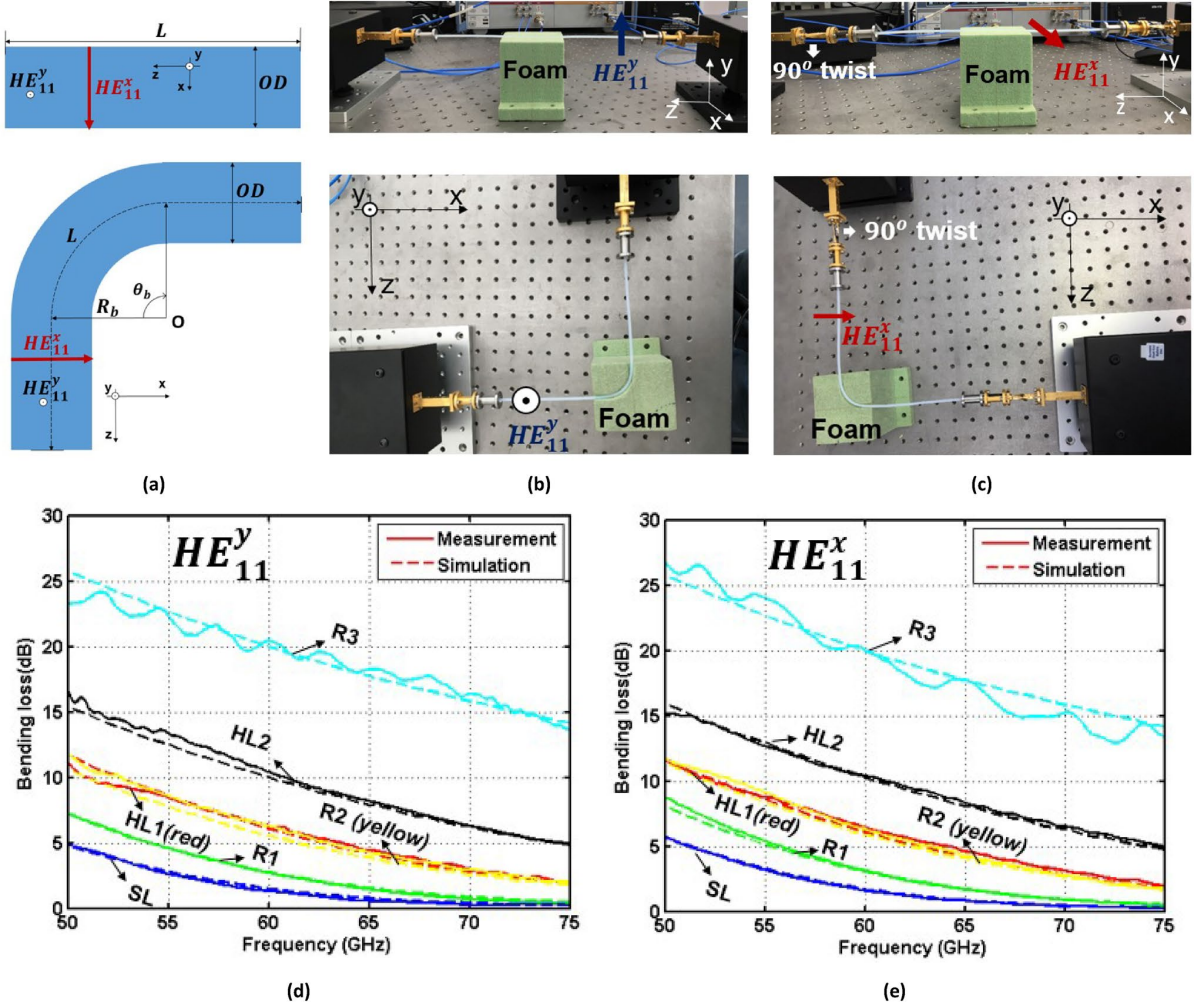
Bending measurement setups and results. **(a)** Cross-section of a straight dielectric waveguide and a bent one with a ${\theta }_{b}$ angle and a ${R}_{b}$ radius of curvature. **(b)** Setups to measure bending loss when ${\theta }_{b}$= 90° and ${R}_{b}$ = 25 mm for the ${HE}_{11}^{y}$ mode. **(c)** For the ${HE}_{11}^{x}$ mode. **(d,e)** Bending loss for the different dielectric waveguides as a function of frequency and for the ${HE}_{11}^{y}$ and ${HE}_{11}^{x}$ modes, respectively. Solid and dashed lines are measured and simulated results, respectively.

Bending effects in dielectric waveguides depend on two main factors: bending radius (${R}_{b}$) and bending angle (${\theta }_{b}$) (see Fig. 2a). In terms of mechanical stress, the numerical simulations carried out with ABAQUS^18^ indicate that deformations of solid and hollow dielectric waveguides (with inner to outer diameters ratio lower than 0.77) are negligible when the waveguides are bent at ${\theta }_{b}$ = 90*°* with ${R}_{b}$ = 15 or 25 mm. As a result, the bending effects have a minor effect on the propagation mode, cutoff frequency and group delay of the waveguides. However, the two fundamental degenerate modes with orthogonal polarizations (${HE}_{11}^{x}$ and ${HE}_{11}^{y}$) in circular dielectric waveguides loose their 90° rotational symmetry under bending configuration. This may lead to different bending effects for each polarization.

### Bending at a 90° angle with a 25 mm radius of curvature

Firstly, all samples were measured to characterize experimentally the bending loss for an angle ${\theta }_{b}$ = 90*°* and a bending radius ${R}_{b}$ = 25 mm, for both the ${HE}_{11}^{y}$ (see Fig. 2b) and ${HE}_{11}^{x}$ modes (see Fig. 2c). Figure 2d,e depict the measured and simulated bending losses of the selected waveguides for the ${HE}_{11}^{y}$ and ${HE}_{11}^{x}$ modes, respectively. Also in this case, a good agreement is observed between simulations and measurements. Thus, it is safe to use simulations to predict the bending loss of dielectric waveguides. In addition, there is no significant difference between the ${HE}_{11}^{x}$ and ${HE}_{11}^{y}$ modes. It is to be observed that the bending losses rise rapidly at lower frequencies because the electromagnetic energy is less confined inside the core of the waveguides and it is more susceptible to being radiated due to bending. Besides, comparing the bending losses between waveguides SL and R3 (or between R1 and R2), it is clear that solid SL (or hollow R1) waveguides are more tolerant than hollow R3 (or R2) ones to bending effects. Indeed, bending losses for the solid waveguide SL are about 0.3 dB in the range 70–75 GHz. This means that the guided mode is very well confined in the waveguide core near the cutoff frequency for the first higher order mode. In contrast, waveguide R3 loses most of the electromagnetic energy by bending.

**Figure 3:**
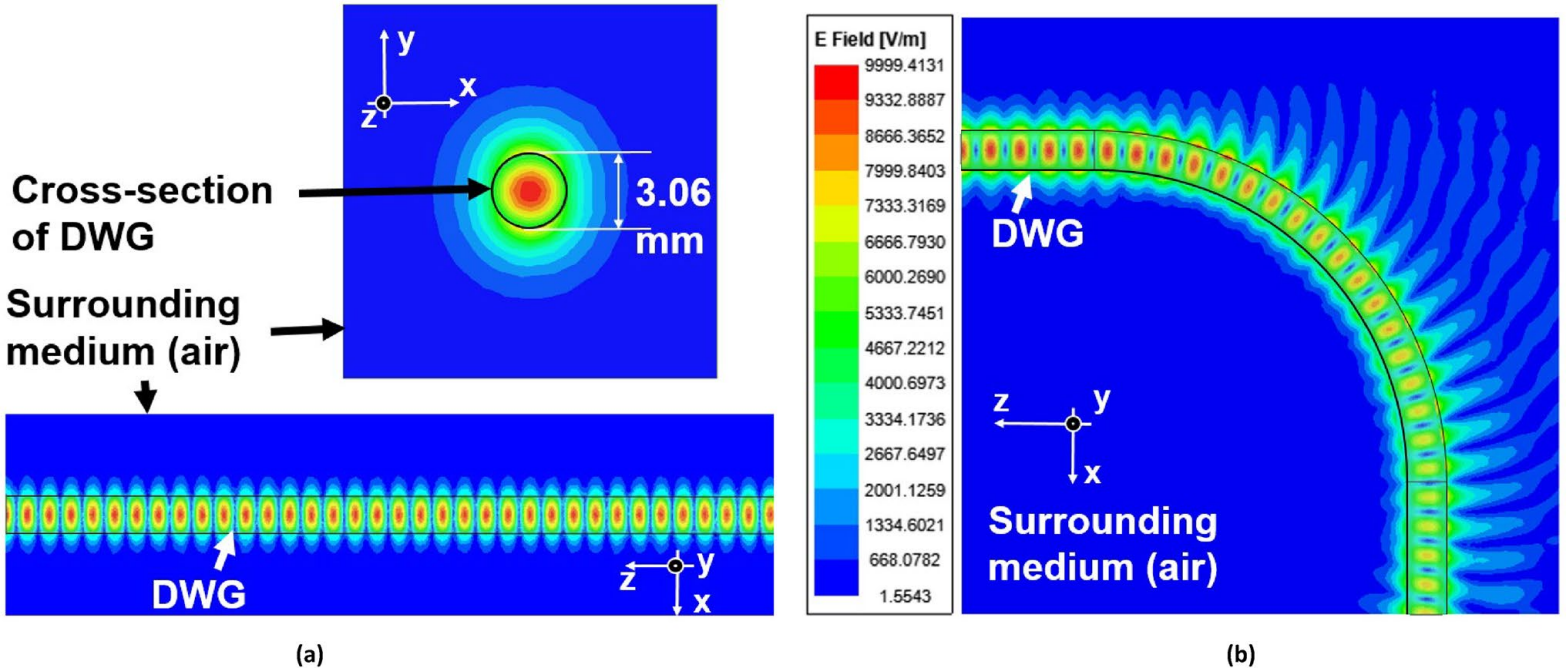
Distribution of the ${HE}_{11}^{y}$ in solid SL waveguide at 60 GHz for two configurations: **(a)** straight and **(b)** bending (${\theta }_{b}$ = 90° and ${R}_{b}$ = 25 mm). The field distributions are simulated results by Ansys HFSS.

Figure 3 illustrates the field distribution at 60 GHz for the ${HE}_{11}^{y}$ mode of solid SL waveguide in the straight and bending configurations (${\theta }_{b}$ = 90° and ${R}_{b}$= 25 mm). It is possible to observe that the guided wave leaks energy in free space when the waveguide is bent. This leakage causes bending losses.

**Figure 4:**
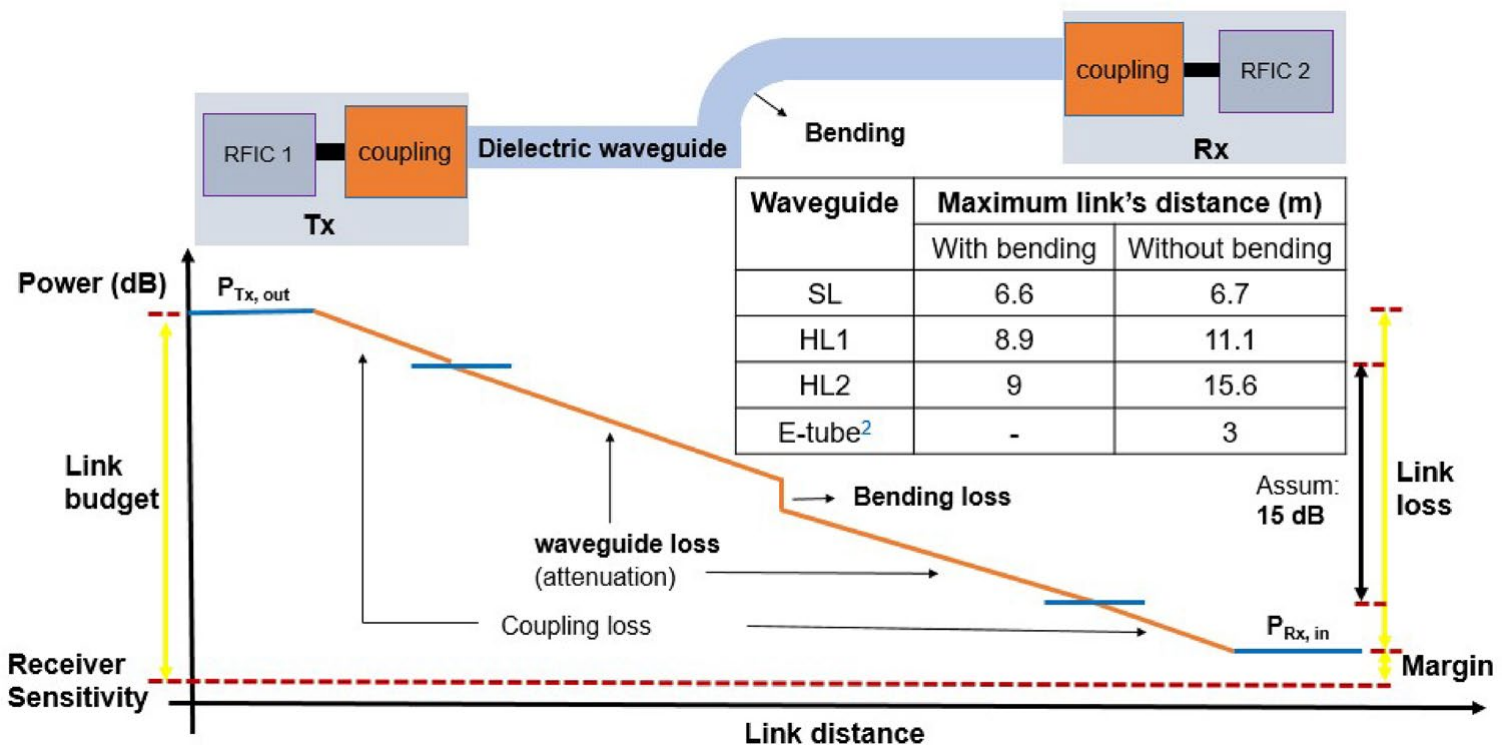
Maximum link’s distance of a wired communication system using bent waveguides SL, HL1 or HL2 (${\theta }_{b}$ = 90° and ${R}_{b}$ = 25 mm). Assume that the link budget includes 15 dB for total losses on the dielectric waveguide.

In order to observe the influence of bending on a link budget, we assume the case of a wired communication system using a radio-frequency (RF) transceiver based on CMOS integrated circuits (ICs). It has been shown^2^ that such set-up can yield a data rate of 25 Gbps with a 70 GHz carrier, provided that the propagation losses in the waveguide are lower than 15 dB (the coupling losses from the IC to the waveguide are excluded from this consideration). Figure 4 represents the maximum link’s distance that could be achieved by using waveguide SL, HL1 or HL2 while respecting the condition of a propagation loss lower than 15 dB in the aforementioned scenario. The maximum transfer distances of solid SL, hollow HL1 and HL2 waveguides reduce by about 1.5, 19.8 and 42.3% due to the bending losses, respectively. However, despite the detrimental effect of bending, waveguides SL, HL1 and HL2 can satisfy the link budget, thus maintaining the quality of the link, for longer distances than the metal-shielded dielectric waveguide E-tube^2^, which is not impaired by bending.

### Bending at different angles

**Figure 5:**
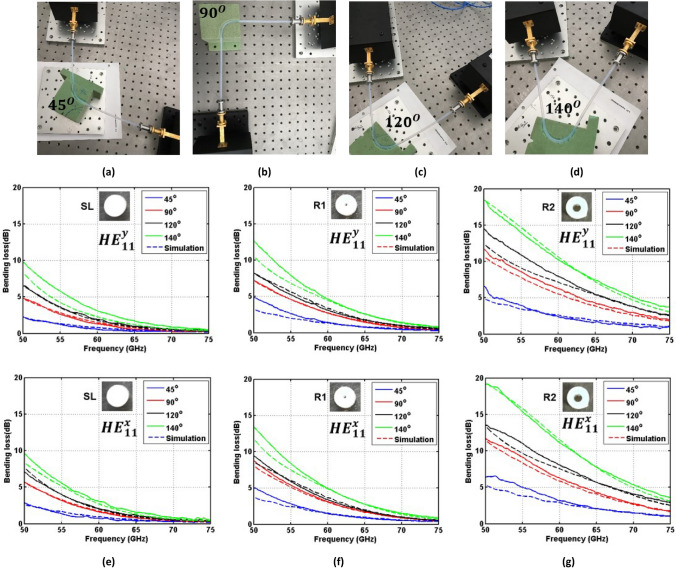
Dielectric waveguide bending configuration for ${R}_{b}$ = 25 mm and different ${\theta }_{b}$ angles: **(a)** 45*°*; **(b)** 90*°*; **(c)** 120*°*; **(d)** 140*°*. **(e–g)** Bending loss of dielectric waveguides SL, R1 and R2 for the ${HE}_{11}^{y}$ and ${HE}_{11}^{x}$ modes, respectively. Solid and dashed lines represent the measured and simulated results, respectively.

Secondly, bending effects were quantified for different angles (45*°*, 90*°*, 120*°* and 140*°*) with a 25 mm radius of curvature (see Fig. 5a–d). Figure 5e-g show the measured bending losses for waveguides SL, R1 and R2, respectively. Each waveguide was tested for the four bending configurations in Fig. 5a–d and for both polarizations. The agreement between the measured and simulated bending losses for the bending angles 45°, 120*°* and 140*°* is not as good as that obtained for a bending angle of 90° and reported in Fig. 2. This is because in simulations, it is tricky to use a rectangular radiation box for dielectric waveguides with bending angles 45°, 120*°* and 140°. However, both measurements and simulations indicate that the bending losses for the two polarizations increase when the bending angle rises due to the smaller power confinement of the modes in the core of the dielectric waveguides. Particularly, Table 2 provides the average bending losses between 50 and 75 GHz for solid SL, hollow R1 and R2 waveguides. Four angles of curvature (45*°*, 90*°*, 120*°* and 140*°*) and the two polarizations (${HE}_{11}^{y}$ and ${HE}_{11}^{x}$ ) are reported for a 25 mm bending radius. In most of the considered cases, the average bending losses for the ${HE}_{11}^{x}$ mode are slightly higher than those for the ${HE}_{11}^{y}$ mode.

**Table 2:** Measured average bending loss (dB) at V-band for different bending angles and a 25 mm radius of curvature.

	${HE}_{11}^{y}$ mode				${HE}_{11}^{x}$ mode			
	45°	90°	120°	140°	45°	90°	120°	140°
SL	0.70	1.46	2.07	3.25	0.85	1.76	2.24	3.14
R1	1.54	2.61	3.05	4.57	1.64	3.08	3.35	4.87
R2	2.44	5.69	7.25	9.47	3.14	5.79	7.39	10.24

### Bending at a 90° angle with a 15 mm radius of curvature

**Figure 6:**
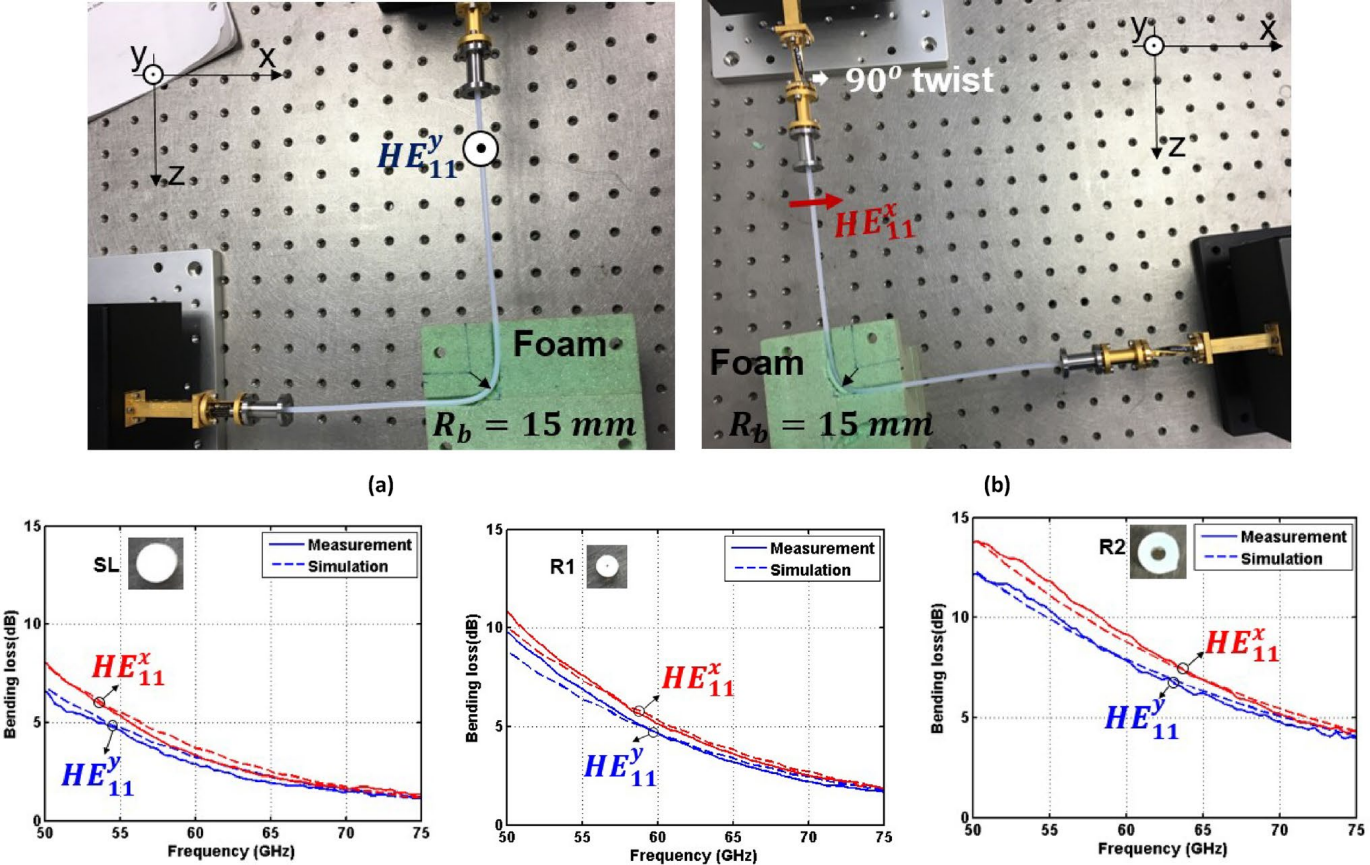
Dielectric waveguides bending configurations with ${R}_{b}$ = 15 mm and ${\theta }_{b}$ = 90*°*. **(a)** For the ${HE}_{11}^{y}$ mode. **(b)** For the ${HE}_{11}^{x}$ mode. **(c–e)** Bending losses corresponding to waveguides SL, R1 and R2, respectively. Solid and dashed lines represent the measured and simulated results, respectively.

Next, bending effects were characterized for a smaller radius of curvature ${R}_{b}$ = 15 mm and a bending angle ${\theta }_{b}$ = 90*°* using dielectric waveguides (SL, R1 and R2) and considering two orthogonal polarizations for the fundamental mode (see Fig. 6a,b). Figure 6c-e show an acceptable agreement between the measured and simulated bending losses of waveguides SL, R1 and R2, respectively. In general, the bending losses rise significantly when the bending radius decreases from 25 to 15 mm. Indeed, with a 15 mm (25 mm) radius of curvature, the average bending losses of waveguides SL, R1 and R2 for the ${HE}_{11}^{y}$ mode are 2.88, 4.48 and 7.40 dB (1.46, 2.61 and 5.69 dB) in the entire V-band. In addition, an apparent difference of the bending effects for the ${HE}_{11}^{y}$ and ${HE}_{11}^{x}$ modes is observed in this case. In waveguides SL, R1 and R2, the bending losses for the ${HE}_{11}^{x}$ mode are higher than for the ${HE}_{11}^{y}$ by about 0.5, 0.5 and 0.97 dB, respectively. This is because the power confinement of the dielectric waveguides for the ${HE}_{11}^{x}$ mode is weaker than the one for the ${HE}_{11}^{y}$ mode when the bending plane is parallel to x-axis.

### Double-bending effects at a 90° angle with a 25 mm radius of curvature

**Figure 7:**
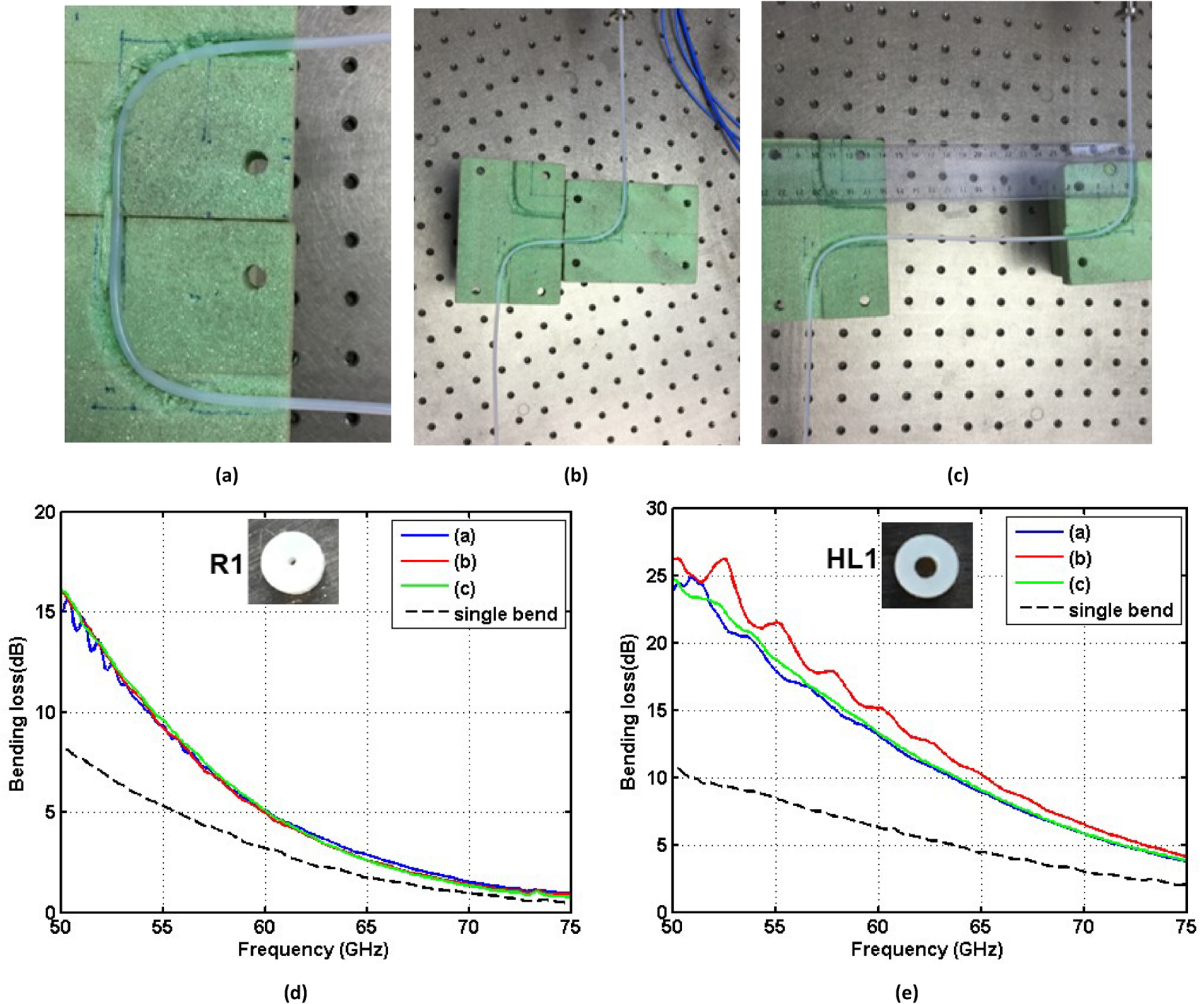
Dielectric waveguides bending configurations with ${R}_{b}$ = 25 mm and ${\theta }_{b}$ = 90*°* for the ${HE}_{11}^{y}$ mode. **(a)** U shape. **(b)** Z shape with two serial bending-points. **(c)** Z shape with 200 mm distance between two bending-points. **(d,e)** Double-bending losses for waveguides R1 and HL1, respectively.

Last but not least, we characterized experimentally the bending losses for different double-bending configurations (U or Z shaped with radius of curvature ${R}_{b}$ = 25 mm and bending radius ${\theta }_{b}$ = 90*°*—see Fig. 7a-c) for the R1 and HL1 dielectric waveguides. This study intends to assess the behavior of dielectric waveguides in a more realistic scenario in which there can exist more than one bending-point to avoid obstacles in the path of our wired link. Figure 7d,e depict the obtained bending losses for waveguides R1 and HL1 and the ${HE}_{11}^{y}$ mode, single bending losses are also shown in the same figure as a reference. These experimental results indicate that the losses in double-bending configurations approximately equal twice the losses in single-bending ones with the same angle and radius of curvature. Therefore, one can just add up the losses due to the different bends in the wired link in order to estimate the total bending losses.

### Bending losses for dielectric waveguides made of different materials

**Figure 8:**
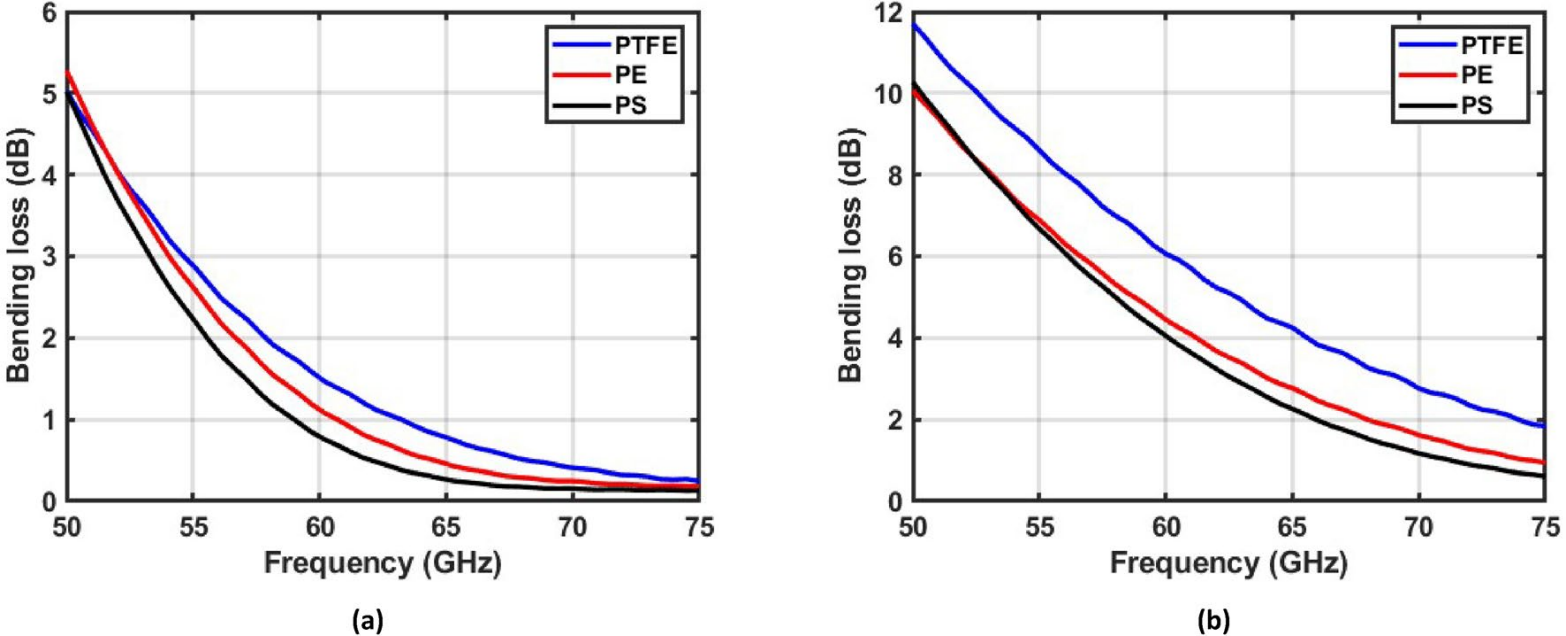
Simulated bending losses of solid **(a)** and hollow **(b)** dielectric waveguides made of materials with different dielectric permittivities. The simulations consider the case of ${HE}_{11}^{y}$ mode propagation and a 90*°* bending angle with a 25 mm radius of curvature.

We also investigated bending losses for solid and hollow dielectric waveguides made of different materials: PTFE—Teflon^15^ (*ε*_*r*_ = 2.03, loss*δ* = 0.0003), PE—Polyethylene^19^ (*ε*_*r*_ = 2.3, loss*δ* = 0.00013) and PS—Polystyrene^20^ (*ε*_*r*_ = 2.53, loss*δ* = 0.001) at 60 GHz (note that the intrinsic properties of plastics are quite stable at millimeter waves^11,12,19^). For each material, we designed one solid and one hollow circular waveguide as shown in Table 3. To compare the impact of the dielectric constants on the bending loss, all PTFE, PE and PS solid waveguides present the same cutoff frequencies (0 GHz for ${HE}_{11}$ mode and 75 GHz for ${TE}_{01}$ mode—the first higher order mode). The hollow waveguides also present similar cutoff frequencies (0 GHz for ${HE}_{11}$ mode and 80 GHz for ${TE}_{01}$ mode) and the same inner to outer diameter ratio (0.37). Note that we used PTFE solid SL and hollow HL1 waveguides as shown in Table 1, which were characterized by measurements and simulations. Figure 8 shows the simulated bending losses of the studied dielectric waveguides for the ${HE}_{11}^{y}$ mode for a 90*°* angle with a 25 mm radius of curvature. It is possible to observe that the bending loss of the waveguides reduces for the dielectric materials with higher dielectric constants.

**Table 3:** Dimensions of dielectric waveguides made of different materials (unit: mm. OD: outer diameter, ID: inner diameter).

	PTFE	PE	PS	Cutoff frequency (GHz)	
				${HE}_{11}$	${TE}_{01}$
Solid	OD = 3.06 (SL)	OD = 2.7	OD = 2.48	0	75
Hollow	OD = 2.94, ID = 1.1 (HL1)	OD = 2.62, ID = 0.97	OD = 2.4, ID = 0.89		80

## Discussion

An experimental characterization of bending effects has been presented for solid and hollow non-shielded dielectric waveguides. The measured characteristics are in good agreement with the simulations, and thus, the simulated results can be safely used to estimate the bending loss in reality. The solid SL and hollow HL1, HL2, R1, R2 and R3 waveguides present in the entire V-band average bending losses for the ${HE}_{11}^{y}$ mode equal to 1.46, 5.65, 9.60, 2.61, 5.69 and 19.10 dB at a 90*°* angle with 25 mm radius of curvature, respectively. The achieved results indicate that the bending losses increase for larger bending angles or for smaller bending radii. Moreover, the bending losses can significantly limit the use of non-shielded hollow dielectric waveguides for energy-efficient links. In contrast, solid waveguides present a good power confinement even when bent. Hence, they are a promising solution for mm-wave links in terms of energy efficiency. However, non-shielded dielectric waveguides are in general sensitive to the external environment. Shielded dielectric waveguides can be investigated to overcome this limitation.

## Methods

### S-parameter measurement

Full two-port measurements of the scattering matrix were carried out in a back-to-back configuration. The experimental setup includes a Rohde & Schwarz VNA (R&S ZVA67), VDI extension modules that cover the 50–75 GHz range with WR-15 output, and WR15-to-circular waveguide transitions with a 4.19 mm inner diameter from SAGE Millimeter (SWT-15165-SB). Besides, a WR-15 90*°* waveguide twist with a UG-385/U Flange operating in the 50-75 GHz range from Pasternack (PE-WR15TW1001) is used to measure the *x*-polarized mode.

### Time gating

During measurements, reflections appeared in the back-to-back configuration. Time gating with a binary bandpass filter in time domain was applied to the measured S-matrix to discard multi-reflections between the various parts of the back-to-back configuration. The smoothing effect of time gating can be appreciated in Fig. 1c.

### Foam—supporting devices

Foam blocks are used to keep the measured dielectric waveguides in place during measurements. Although foam (*ε*_*r*_ ≈ 1^10,21^) has a negligible impact on the cutoff frequency and group delay of the waveguides, it may increase the transmission loss. For this reason, the same material and blocks with similar dimensions were used both for straight and bending configurations.
